# The mediating role of flow in the relationship between simulation design and simulation educational satisfaction in korean nursing students: a cross-sectional study

**DOI:** 10.1186/s12912-024-01946-5

**Published:** 2024-04-25

**Authors:** Eun-Kyung Lee, Eun-Joo Ji

**Affiliations:** 1 College of Nursing, The Research Institute of Nursing Science, Daegu Catholic University, Daegu, Republic of Korea; 2 Department of Nursing, College of Medicine, Catholic Kwandong University, 24, Beomil-ro 579beon-gil, Gangeung-si, 25601 Gangwon-do Republic of Korea

**Keywords:** Education, Mediation analysis, Nursing students, Simulation Training

## Abstract

**Background:**

In Korea, there has been recent interest in nursing simulation education. In nursing, simulation education has many advantages, such as improving nursing students’ problem-solving and judgment skills. Simulation education satisfaction is an indicator for evaluating educational performance from the learners’ perspective and an important criterion for the development and progress of nursing education. Therefore, based on NLN/Jeffries simulation theory, this study aims to identify the relationship between simulation design and educational satisfaction and to confirm the mediating effect of flow.

**Methods:**

This cross-sectional study was conducted using 143 fourth-year nursing students who had participated in classes using simulations at three universities in Seoul, Daegu, and Jeonbuk. Data were collected from April 24 to May 3, 2023. Demographic data, simulation design scale (SDS), flow in simulation, and the educational satisfaction scale in simulation were collected via an online questionnaire. The collected data were analyzed through t-test, ANOVA, Scheffé test, and Pearson’s correlation coefficient using SPSS 25.0. The mediating effect of flow was analyzed through the three-stage mediation effect procedure using hierarchical regression analysis and the Sobel test.

**Results:**

The simulation educational satisfaction had a statistically significant positive correlation with simulation design (*r* = .65, *p* < .001) and flow (*r* = .47, *p* < .001), and simulation design was positively correlated with the flow (*r* = .55, *p* < .001). The simulation design had a statistically significant effect on flow, which was the mediating variable (β = 0.55, *p* < .001). Additionally, simulation design had a statistically significant effect on simulation educational satisfaction (β = 0.56, *p* < .001). The significance of the mediating effect of flow on the relationship between simulation design and simulation educational satisfaction was investigated using the Sobel test, and the mediating effect of flow was found to be statistically significant (Z = 5.36, *p* < .001).

**Conclusion:**

The significance of the current study lies in its confirmation of the link between simulation design and simulation educational satisfaction, as well as the mediating function of flow. Nursing students can achieve simulation educational satisfaction through simulation-based education if simulation educators follow best practices that improve flow through well-organized simulation design.

## Background

Simulated education is an integrated process of knowledge, skill, and experiential learning for nursing students and is a teaching and learning strategy to facilitate nursing competencies [1]. The National Council of State Boards of Nursing recommends simulation use in nursing education since it can safely replace up to half of clinical practice training hours [2]. The Korean Accreditation Board of Nursing Education also allows simulation-based education to replace four credits of clinical practice credits in the third phase and up to six credits in the fourth phase [3].

Simulation-based education requires instructor preparation and consideration, from setting learning objectives to developing scenarios, preparing supplies, prebriefing, standardized patient training, preparing the simulator, running the simulation, and debriefing [4], making it an education method with a high instructor burden. Nevertheless, simulation-based education improves nursing students’ problem-solving, clinical judgment, and communication skills [5, 6] and develops the teamwork necessary for clinical nurses [7], leading to its increasing use in nursing education.

However, simulation-based education does not only bring the aforementioned positive outcomes to participating students. Studies on nursing students in Korea have reported that nursing students who participated in simulation-based education felt nervous and anxious, overwhelmed and embarrassed, and guilty about making mistakes [5, 8, 9]. Stress and low self-confidence due to simulation-based education [10] and the idea that others are watching them lead to anxiety, resulting in passivity when participating in simulations with peers [11]. Additionally, high anxiety levels experienced during the simulation process can drive learners into a panic, which leads to negative learning effects and decreases simulation educational satisfaction [12, 13]. Since simulation educational satisfaction is a variable that is evaluated to establish simulation design and educational strategies and to check the outcome of the simulation experience (i.e., the performance of simulation-based education from the learner’s perspective) [14], it is necessary to identify factors related to simulation educational satisfaction.

The NLN/Jeffries theory of simulation [14] explains that within the overall context of simulation-based education, simulation design influences the simulation experience, resulting in simulation-based education outcomes. Since the simulation experience is influenced by the dynamic interaction between the instructor/instructional strategy and the learner, simulation-based education design, which includes the learning objectives, simulation contents, and the instructor’s cueing and debriefing, can influence simulation outcomes such as learning satisfaction [14]. During the debriefing process, learners who participated in the simulation acquire knowledge and skills about the simulation situation and can learn through reflection [7]. Well-designed simulation-based education improves clinical decision-making, promotes learning engagement, and increases satisfaction and confidence in learning [15–17]. Adequate design is essential for simulation-based education.

Flow is a state of absolute immersion in a specific activity to the point of forgetting the time and activity, and learners in a flow state are so focused on the task at hand that they believe the learning activity is happening on its own as there is no separation between the learning activity and their perception [18]. This highlights the value of the flow experience in enhancing intrinsic motivation in remote learning, which depends on learners’ self-direction, and in producing excellent learning outcomes [19]. Since simulation-based education involves learning in a hypothetical situation, learner flow is even more important, as learners in simulations report feeling like they are being observed by someone else [11] and that it is not real [7], indicating that they may not be able to immerse themselves in the simulation situation [18]. If learners are not immersed in the simulation, their ability to achieve learning outcomes is not only affected but can also lead to anxiety, which can hamper their collaboration with their peers [11]. This suggests that a simulation design that increases physical, conceptual, and psychological fidelity is necessary to induce flow, as flow increases students’ motivation, enjoyment, and satisfaction with the learning process.

Therefore, to maximize the effectiveness of simulation-based education, instructors should develop strategies to increase flow in simulation situations to elicit learners’ flow experiences [15, 20]. In recent years, many studies on simulation education in Korea have identified learning outcomes and defining factors after training [5–7, 9, 12, 20]. However, there is a lack of research on the role of flow in learning design and simulation educational satisfaction.

Therefore, this study aims to investigate the relationship between simulation design, which is classified as a key factor in simulation education, and simulation educational satisfaction [14] among nursing students with simulation experience and to identify the mediating effect of flow in the relationship between the two variables, thereby providing a basis for developing measures to increase simulation educational satisfaction.

The study seeks to investigate:

1. The levels of simulation design, flow, and simulation educational satisfaction perceived by participants are identified.
2. The differences in simulation educational satisfaction based on participants’ general characteristics are identified.
3. The correlations among simulation design, flow, and simulation educational satisfaction are identified.
4. The mediating effect of flow on the relationship between simulation design and participants’ simulation educational satisfaction is identified.

## Methods

### Design

The study used a cross-sectional design. This descriptive survey study investigates factors related to simulation educational satisfaction among nursing students.

### Participants

This study included nursing students from three universities in Korea. The study’s participant selection criteria were based on convenience sampling. To be eligible to participate in this study, the participant must be a fourth-year nursing student who has taken part in simulation-based programs, complete cognitive and behavioral competency, has the ability to provide informed consent, and has voluntarily agreed to participate in the survey. All nursing students who fulfilled the study’s inclusion criteria were invited to participate in the optional survey. The exclusion criteria were on leave from school, or diagnosed with a significant mental illness or psychiatric disorder.

A sample size of 136 was required after calculating the number of participants in the study using the G-power 3.1 program with an effect size of 0.15, a power of 0.90, and a significance level of 0.05 for regression analysis with eight expected independent variables. With an inappropriate response rate of approximately 10%, 152 participants were selected for the study. A total of 152 people responded, and 143 were selected for the final analysis after excluding nine people who gave biased responses.

### Instruments

#### Simulation educational satisfaction

Simulation educational satisfaction was measured using the Educational Satisfaction Scale in Simulation for Nursing Students by Kim and Heo [8]. The scale consisted of three factors with sixteen items: learning content (six items), situational competency (six items), and emotional response (four items), scored on a 5-point Likert scale ranging from 1 point (*strongly disagree*) to 5 points (*strongly agree*). Scores were calculated by summing the scores for each item. The total score ranged from 16 to 80, with a higher score indicating a higher satisfaction level with simulation-based education for nursing. At the time of scale development, the reliability of Cronbach’s α was 0.89. In this study, Cronbach’s α was 0.95.

#### Simulation design

Simulation design was measured using the Simulation Design Scale (SDS) developed by the American Nurses Association [21], adapted to Korean, and validated by Yoo and Kim [20]. It has five factors: goals and information (six items), support (four items), problem-solving (five items), feedback/guided reflection (four items), and fidelity (two items). The items were scored on a 5-point Likert scale ranging from 1 point(*strongly disagree*) to 5 points (*strongly agree*). Scores were calculated by summing the scores for each item. The total score ranged from 21 to 105, with a higher score indicating a more adequate simulation design. At the time of scale development, the reliability of the scale, Cronbach’s α was 0.92. Cronbach’s α was 0.90 in the study by Yoo and Kim [20] and 0.94 in this study.

#### Flow

Flow was measured with a 10-item scale that was adapted by Yoo and Kim [20] from a simplified flow measure developed by Engeser and Rheinberg [22] to measure the degree of flow in simulation education, adding “during simulation” to each item and excluding items measuring antecedents of flow. Each item was scored on a 5-point Likert scale ranging from 1 point (*strongly disagree*) to 5 points (*strongly agree*). Scores were calculated by summing the scores for each item. The total score ranged from 10 to 50, with a higher score indicating a higher level of flow in simulation-based education. At the time of scale development, the reliability of Cronbach’s α was 0.92. Cronbach’s α was 0.84 in the study by Yoo and Kim [20] and 0.84 in this study.

### Data collection and ethical considerations

Data collection was conducted using a web-based survey after approval from the Institutional Review Board of the author’s institution (IRB No. 23-01-0102). The data were collected from April 24 to May 3, 2023.

The survey was conducted by contacting professors at one university in each of the Seoul, Daegu, and Jeonbuk regions by phone to ensure that the students had experience with simulation, explaining the purpose and methodology of the study, and asking for their cooperation. The recruitment document and study description were delivered through the fourth-year class representative.

Potential participants were given the URL for the survey that included information about the study’s objectives, the questionnaire procedure, anonymity of consent forms, and information regarding the voluntary withdrawal from the survey without consequence. Participants received information on data confidentiality: they were assured that the use of their data would follow personal information protection guidelines and that the data they provided would be deleted after the research. Participants who gave their consent prior to filling out the questionnaire were all included; those who did not were excluded. Participants were only allowed to complete the survey if they read the consent form within the URL survey and checked the I agree box.

The web-based survey was conducted through a specialized survey site. To prevent duplicate survey participation, only one response could be submitted per IP, and only one response per item was allowed to exclude multiple responses. The mobile phone numbers collected for sending rewards were used to check for duplicate participation.

The data collected through the survey was anonymized with a unique identification code and deleted immediately after saving the research data. Stored research data was encrypted and would be permanently deleted three years after the completion of the study. Participants’ mobile phone numbers that were collected to provide rewards were permanently deleted immediately after the rewards were sent.

### Data analysis

The collected data were analyzed using the SPSS 25.0 program.

1) The general characteristics of the participants, simulation design, flow, and simulation educational satisfaction were calculated as frequencies and percentages and means and standard deviations.

2) Differences in simulation educational satisfaction according to the general characteristics of the participants were analyzed by *t*-test and one-way ANOVA, and Sheffe’s test was used as the post hoc test.

3) The relationship between simulation educational satisfaction, simulation design, and flow was analyzed by Pearson’s correlation coefficient.

4) The mediating effect of flow on the relationship between simulation design and simulation educational satisfaction was examined using simple and multiple regression analyses based on the methodology of Baron and Kenny’s three-step procedure. The significance of the mediating effect was confirmed by the Sobel test.

## Results

### General characteristics

The mean age of the participants was 22.66 years (SD = 2.11). Most participants (90.9%, *n* = 130) were under 25 and women (85.3%, *n* = 122). More than half (78.8%, *n* = 84) had an academic grade of 3.5 or higher. Seventy-two preferred a lecture class. 110 subjects responded that they were satisfied with their nursing major and clinical practice (Table 1).

**Table 1:** Differences in simulation education satisfaction according to general characteristics (*N* = 143)

Characteristics	Categories	N (%)	M ± SD	t/F/r Scheffe	*p*
Age (years)	< 25	130(90.9)	3.93 ± 0.43	0.96	0.384
	25-<30	9(6.3)	4.05 ± 0.47		
	30–31	4(2.8)	3.69 ± 0.64		
Gender	Female	122(85.3)	3.95 ± 0.42	1.33	0.187
	Male	21(14.7)	3.82 ± 0.50		
Academic	< 3.0	16(11.2)	3.88 ± 0.53	2.23	0.087
achievement	3.0∼<3.5	43(30.1)	3.83 ± 0.38		
	3.5-<4.0	55(38.5)	4.04 ± 0.41		
	≥ 4.0	29(20.3)	3.90 ± 0.45		
Satisfaction of	Very satisfied^a^	26(18.2)	4.33 ± 0.39	15.29	< 0.001
major	Satisfied^b^	84(58.7)	3.87 ± 0.36	d < b,c < a	
	Moderate^c^	29(20.3)	3.85 ± 0.42		
	Unsatisfied^d^	4(2.8)	3.22 ± 0.42		
Satisfaction of	Very satisfied^a^	22(15.4)	4.31 ± 0.41	13.75	< 0.001
clinical practice	Satisfied^b^	88(61.5)	3.92 ± 0.35	d < a,b, c	
	Moderate^c^	29(20.3)	3.78 ± 0.45		
	Unsatisfied^d^	4(2.8)	3.17 ± 0.36		
Preferred learning	Lecture	72(50.3)	3.88 ± 0.46	1.34	0.266
style	Discussion	14(9.8)	3.90 ± 0.35		
	Practice	57(39.9)	4.00 ± 0.41		

### Degree of variables

The mean of simulation educational satisfaction, simulation design, and flow scores were established as 3.93 (SD = 0.43), 4.17 (SD = 0.45), and 3.73 (SD = 0.53), respectively. There was a significant relationship between simulation educational satisfaction and simulation design (*r* = .65, *p* < .001) and flow (*r* = .47, *p* < .001). There was also a significant relationship between flow and simulation design (*r* = .55, *p* < .001) (Table 2).

**Table 2:** Degrees of variables (*N* = 143)

Variables	M ± SD	Item mean ± SD
Simulation education satisfaction	62.90 ± 6.93	3.93 ± 0.43
Learning content	25.85 ± 2.93	4.31 ± 0.49
Situational competency	25.34 ± 2.92	4.22 ± 0.49
Emotional response	11.71 ± 3.77	2.93 ± 0.94
Simulation design	87.50 ± 9.40	4.17 ± 0.45
Educational goal and contents	24.69 ± 3.00	4.12 ± 0.50
Support	16.41 ± 2.28	4.10 ± 0.57
Problem-solving	20.94 ± 2.44	4.19 ± 0.49
Feedback	17.29 ± 2.04	4.32 ± 0.51
Fidelity	8.17 ± 1.33	4.09 ± 0.67
Flow	37.30 ± 5.30	3.73 ± 0.53

### Differences in simulation educational satisfaction according to general characteristics

The differences in simulation educational satisfaction according to the general characteristics of participants are shown in Table 1. There were statistically significant differences in simulation educational satisfaction in satisfaction of major (F = 15.29, *p* < .001) and satisfaction of clinical practice (F = 13.75, *p* < .001) (Table 1).

### Correlation among simulation educational satisfaction, simulation design, and flow

The simulation educational satisfaction of the participants was positively correlated with simulation design (*r* = .65, *p* < .001) and flow (*r* = .47, *p* < .001), and simulation design was positively correlated with the flow (*r* = .55, *p* < .001) (Table 3).

**Table 3:** Correlations among variables (*N* = 143)

Variables	Simulation education satisfaction	Simulation design
	r (p)	r (p)
Simulation education satisfaction	1	0.65 (< 0.001)
Simulation design	0.65 (< 0.001)	1
Flow	0.47 (< 0.001)	0.550 (< 0.001)

### The mediating effect of flow on the relationship between simulation design and simulation educational satisfaction

**Fig. 1:**
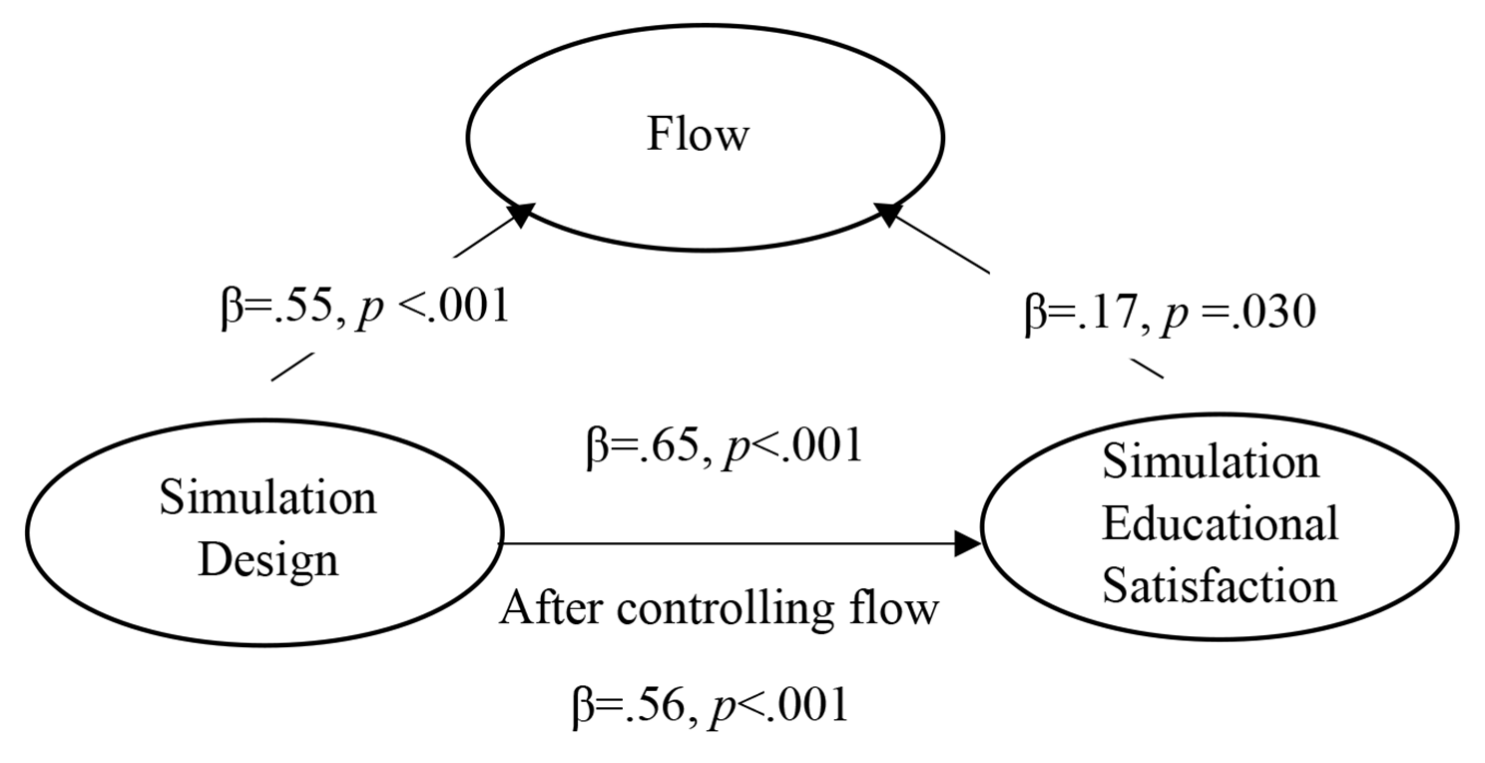
The mediating effect of flow on the relationship between simulation design and simulation educational satisfaction

The results of assessing the mediating effect of flow on the relationship between simulation design and simulation educational satisfaction of the participants were as follows (Table 4; Fig. 1). As a result of checking for multicollinearity among the independent variables to verify the assumptions for the regression analysis, the tolerance was 0.70, and the variance expansion factor was 1.43, indicating no multicollinearity. As a result of checking the autocorrelation of the dependent variable, the Durbin-Watson value was 2.06, which was close to 2, indicating that they were independent of each other. Therefore, a regression analysis was conducted.

The mediation analysis was conducted in three steps, as follows: Step 1 analysis showed that the independent variable, simulation design, had a significant effect on the dependent variable, simulation educational satisfaction (β = 0.65, *p* < .001). Step 2 analysis showed that the independent variable, simulation design, had a significant effect on the mediator variable, flow (β = 0.47, *p* < .001). Step 3 analysis showed that the mediator variable, flow, had a significant effect on simulation educational satisfaction while controlling for the independent variable, simulation design (β = 0.17, *p* = .003). Controlling for the influence of the mediator variable, flow, the effect of the independent variable, simulation design, on simulation educational satisfaction decreased compared to Step 1, where the influence of the mediator variable was not considered but was still statistically significant (β = 0.56, *p* < .001), confirming the partial mediating effect of flow.

**Table 4:** The mediating effect of flow on the relationship between simulation design and simulation educational satisfaction (*N* = 143) SD = Simulation design; SES = Simulation educational satisfaction

Variables	B	SE	β	t	*p*	R^2^	F	*p*
1. SD → SES	0.63	0.06	0.65	10.09	< 0.001	0.42	101.81	< 0.001
2. SD → Flow	0.65	0.08	0.55	7.81	< 0.001	0.30	60.99	< 0.001
3. SD, Flow → SES						0.44	54.67	< 0.001
SD → SES	0.54	0.07	0.56	7.34	< 0.001			
Flow → SES	0.14	0.06	0.17	2.19	0.030			
Sobel test: Z = 5.36, *p* < .001								

In other words, there was a direct effect of simulation design on simulation educational satisfaction and an indirect effect of simulation design on simulation educational satisfaction via flow. The model had an explanatory power of 44.0% and was statistically significant (F = 54.67, *p* < .001). Using the hierarchical regression analysis proposed by Baron and Kenny [23] to test the mediating effect as well as the Sobel test to confirm the significance of the mediating effect, a partial mediating effect of flow was identified in the relationship between simulation design and simulation educational satisfaction (Z = 5.36, *p* < .001).

## Discussion

Assessment of simulation educational satisfaction is an important process in the development and advancement of simulation-based nursing education [24]. Accordingly, this study explored the relationship between simulation design, flow, and simulation educational satisfaction among nursing students with simulation education experience. As a result of the study, simulation design was correlated with simulation educational satisfaction, and flow had a partial mediating effect between the two variables. A discussion of the findings was as follows:

The simulation educational satisfaction score in this study was 62.90 ± 6.93, which was higher than the scores of 57.12 ± 8.21 [8] and 57.26 ± 6.53 [17] in previous studies using the same scale. The higher the satisfaction with the major, the higher the satisfaction with clinical practice [25], and the higher the satisfaction with university life, the higher the simulation educational satisfaction. In a previous study, only 47.5% responded that they were satisfied with their university life [8]. However, in this study, 76.9% were satisfied with their major and clinical practice, indicating that simulation educational satisfaction was higher than in the previous study. Furthermore, in this study, the more satisfied students were with their majors and clinical experiences, the higher their simulation educational satisfaction was, supporting this inference. When examining the mean ratings for each of the simulation educational satisfaction subfactors, the emotional response score was the lowest in both this study and the previous study [8]. This may have been because simulation-based education caused nursing students to feel nervous, anxious, or embarrassed [9, 26, 27]. Student feelings assume a fundamental part in comprehension that relates to the securing and moving of information and clinical abilities [28]. As psychological well-being plays an important role in achieving learning outcomes in nursing education using simulation [29], efforts should be made to reduce the stress, anxiety, tension, and pressure experienced by nursing students during simulation to increase their simulation educational satisfaction.

In this study, nursing students were deeply engaged in the simulation and satisfied with their simulation-based education when the simulation design was adequate. The Healthcare Simulation Standards of Best Practice^™^ by the international nursing association for clinical simulation and learning (INACSL) enhance simulation science, communicate best practices, and offer evidence-based recommendations for the use and creation of a thorough standard of practice [4]. The standards explain that simulation design influences simulation experience within the overall context of simulation education [4], which supports the findings of this study. Several studies with domestic and international nursing students have demonstrated that the better the simulation design, the higher the flow and satisfaction [15, 17, 30], suggesting that simulation design is critical to achieving positive outcomes in simulation-based education. The average score of 4.17 ± 0.45 was greater than the results of several studies on Korean nursing students [12, 16, 20, 29]. However, it is less than the 4.54 ± 0.38 score in a study conducted abroad [31]. Thus, it suggests that Korean nursing education is moving toward simulation-based instructional design.

For the sub-factor scores of simulation design, the feedback score was the highest, and the fidelity score was the lowest, and a study of Korean nursing students reported similar results [29]. However, the Norwegian nursing students had the highest fidelity score [31]. Fidelity is categorized into physical, conceptual, and psychological fidelity, and fidelity is an important factor in simulation design because realism immerses learners in the simulation situation. Visual stimulation is very important because it can increase learners’ interest in learning and their retention ability [32]; however, the appearance of the simulator may have affected the realism because the high-fidelity equipment used in domestic nursing education is made overseas. In such an environment, to increase physical, conceptual, and psychological fidelity, professors are expected to increase overall fidelity by developing scenarios with realism in consultation with clinical experts [33]; using a variety of moulages that can embody the patient’s current condition or features; using technologies that provide realistic graphics, sounds, haptics, or virtual reality appropriately; and providing tips such as examples, prompts, hints, and explanations to support the learning process.

The support score was the second lowest among the sub-factors of simulation design, indicating that students were not receiving help from their instructors when they needed it during the simulation. In a study of Korean nursing students, support scores were among the lowest [15, 29], supporting the findings of this study. This can be interpreted in the same vein as the low score of emotional response among simulation educational satisfaction, suggesting that participants experienced negative emotions such as stress when they did not receive adequate support during the simulation. In simulation-based education, learning is achieved through self-reflection, so the importance of a psychologically safe environment is emphasized throughout the entire simulation process, from prebriefing to debriefing. INACSL has also added professional development and prebriefing: preparation and briefing to its revised 2021 healthcare simulation standards of best practice to create a psychologically safe environment [4]. Therefore, by utilizing the INACSL standards in simulation-based education, simulation instructors can create a supportive environment where students in simulations receive help when they want it and where teachers can quickly see what students need and help them with it, which in turn will improve flow and simulation educational satisfaction.

The similarity in the ranking of simulation design sub-factor scores in this study and previous studies suggests that efforts are needed to strengthen the simulation design capabilities of Korean nurse educators. To increase fidelity, simulation technology support should be provided at the school level. In addition, since simulation is a recently utilized teaching method in Korean nursing education, most nurse educators lack experience in simulation education. This lack of experience may have limited their ability to understand students’ experiences in simulation situations, which, in turn, affected their ability to provide students with the support they needed during simulation.

The INACSL guidelines, revised in 2023, added professional development, suggesting that instructors should strive to provide high-quality simulation education that reflects the needs of learners with new knowledge related to simulation [4]. Therefore, in addition to theoretical preparation for professional development before starting simulation education, it seems necessary to participate in practical training to experience the role of learner and educator.

The association between simulation design and educational satisfaction in the model was partially mediated by the mediating variable, which was the flow of the simulation. In other words, simulation design has a direct effect on simulation educational satisfaction, and an indirect effect on nursing students’ simulation educational satisfaction by affecting the flow of the simulation. This means that if the learning objectives and content are well set, a supportive simulation environment is created where learners can immerse themselves in each situation to discover and solve problems, and simulation-based education is designed to provide appropriate feedback, simulation educational satisfaction can be increased. The more immersive the simulation class, the greater the satisfaction with simulation-based education. Studies have shown that flow has a partial mediating effect between VR simulation design and educational satisfaction [17] and between simulation design and satisfaction and self-confidence [18]. The simulation design seemed to induce learners to flow into the simulation, which increased their simulation educational satisfaction because they performed well once they engaged in the simulation activity.

Despite the strengths of this study, the study also has some limitations. This study used a cross-sectional design, which can only capture the associations between variables at one point in time. Other observational studies are needed to clarify the relationships between variables. Therefore, understanding the causality of the relationships between variables requires further studies. The survey was carried out at three Korean universities using a non-probability convenience sampling design, which calls into question the generalizability of the findings. A large-scale study on nursing students from Korean universities in different cities and regions could provide better insight and generalizable findings on simulation satisfaction in Korean universities. Additionally, self-reporting surveys may produce skewed, exaggerated, or understated results influenced by social desirability.

## Conclusion

This study analyzed the relationship between simulation design, flow, and simulation educational satisfaction based on NLN/Jeffries simulation theory [14] and previous studies to identify factors related to nursing students’ simulation educational satisfaction. The study revealed that simulation design, flow, and simulation educational satisfaction are positively related and that flow has a partial mediating effect on the relationship between simulation design and simulation educational satisfaction. The results suggest that immersion in simulation situations plays a mediating role in the relationship between simulation instructional design and simulation training satisfaction. To increase the simulation educational satisfaction, the scale should be designed by considering the learning goals and contents, support methods during simulation, problem-solving methods, feedback, realism, and building an immersion strategy. This study makes the following suggestions. First, further research should assess various variables suggested by the NLN/Jeffries simulation theory. Second, it is necessary to develop faculty development programs to improve the ability to apply simulation design and teaching strategies and to determine their effectiveness. Third, research needs to facilitate learner flow in simulation-based education.

## Data Availability

Availability of data and materials. The datasets used and/or analyzed during the current study are available from the corresponding author upon reasonable request.

## Abbreviations

INACSL: International nursing association for clinical simulation and learning
SDS: Simulation Design Scale
